# Herbal Medicine in Acute and Chronic Sinusitis; Still a Cinderella?

**DOI:** 10.1007/s11882-025-01195-9

**Published:** 2025-03-11

**Authors:** Alexios Vardouniotis, Maria Doulaptsi, Georgia Liva, Ioannis Vlastos, Alexander Karatzanis, Emmanuel Prokopakis

**Affiliations:** 1 ENT Consultant, NHS Treatment Center, Portsmouth, UK; 2 Department of Otorhinolaryngology, University of Crete School of Medicine, Heraklion, Crete Greece; 3 Department of Otorhinolaryngology, Aghia Sophia Children’s Hospital, Athens, Greece

**Keywords:** Herbal medicine, Phytoneering, Acute rhinosinusitis, Chronic rhinosinusitis, Herbal extract

## Abstract

**Purpose of Review:**

To set the context of current knowledge regarding the role of herbal medicine in acute and chronic rhinosinusitis treatment.

**Recent Findings:**

It is estimated that adults experience 1–3 episodes of viral rhinosinusitis per year and this number increases up to 8–10 episodes in preschool children. The symptoms of acute rhinosinusitis tend to significantly overlap with symptoms of other upper respiratory infections, making the diagnosis quite difficult. The division of rhinosinusitis into bacterial or non-bacterial is clinically important in order to determine appropriate treatment and the administration of antibiotic treatment. Treatment of acute rhinosinusitis is symptomatic and includes nasal rinsing, decongestants, corticosteroids, and combinations of the above. Herbal medicine has been traditionally underestimated in Western world. Nowadays, however, treatment of diseases with the use of medicinal plant treatments is gaining more and more followers. In this context, certain herbal extracts have been tested for viral, post- viral and chronic rhinosinusitis.

**Summary:**

Phytoneering is an innovative pharmaceutical technique in research and production of herbal medicines. Herbal extracts produced with phytoneering vary in quality and active substances. In terms of quality, safety and efficacy, herbal medicines are at least on par with synthetically produced medicines, having significantly less unwanted side effects. Certain herbal extracts have been tested, and in cases of acute rhinosinusitis are effective. In chronic sinusitis those extracts show promising results and might prove a good alternative without side effects.

## Introduction

Acute rhinosinusitis (ARS—acute rhinosinusitis) refers to the inflammation of the nose and paranasal sinuses and is characterized by the existence of specific symptoms and signs, being one of the most common reasons for GP (general practioner) visits. The estimated incidence of ARS ranges from 1.39% to 9% per year depending on the study method and the population included, being responsible for 2–10% of visits to primary health facilities and otolaryngology clinics [1–3]. In chronic rhinosinusitis (CRS), symptoms are present for over 12 weeks presenting as a multifactorial disease that affects more than 10% of the population [1].

It is well known that ARS can be caused by both viral and bacterial pathogens; however, in most cases symptoms usually start with a viral infection [1]. The frequency of acute bacterial rhinosinusitis (ABRS) is unknown, but is estimated at 0.5–2.0% of all ARS [1, 3, 4]. The division of ARS into bacterial or non-bacterial is clinically important to determine further treatment and in particular the administration of antibiotics. Unfortunately, excessive and inappropriate use of antibiotics, not only by patients but by health care providers also, has led to a significant socioeconomic burden [1].

In one of the very few prospective observational studies regarding ARS, researchers in Scandinavia determined the direct cost of an episode to be 266 euros [5]. Recently, the indirect cost of an episode in Spain was estimated to be between 224 and 439€ depending on the treatment method [6]. Assuming that patients will be absent from work during sickness, indirect costs increase from 747$ to 820$, depending on whether or not a course of antibiotics is prescribed [1].

The World Health Organization (WHO) defines herbal medicine as a method of treatment including herbs, herbal materials, herbal preparations and complete herbal products that contain as active ingredients parts of plants, other plant materials or combinations. During the history of mankind people have depended on nature to cure illness and even today herbal medicines constitute a major treatment pylon in developing countries.

Western medicine has been prejudiced against herbal medicine for a long time, even in the context of primary healthcare system. Such treatment was not officially recommended, was not considered first line treatment, was given as add on treatment in certain patients, and believed to act as “placebo”. Over the years modernization on the selection and preparation of herbal medicines alongside with accumulating knowledge on their safety and effectiveness, has led to increased utilization of herbs in developed countries.

Phytoneering, as a term, stands for the utilization of innovative pharmaceutical techniques and processes in the research and production of herbal medicines [7, 8], that have been used to treat upper respiratory tract infections (URTI) [9]. Based on data from preclinical and clinical studies, certain products have been proven effective in treating ARS and show promising results for CRS [10, 11]. Since treatment of ARS is symptomatic, specific herbal extracts are a safe and equivalent alternative to traditional pharmaceutical products [7–11].

## Definitions

EPOS2020 (European Position Paper on Rhinosinusitis), defines ARS in adults as an inflammation of the nose and paranasal sinuses that can last up to 4 weeks and is accompanied by a sudden onset of symptoms [1]. This definition is based mainly on expert opinion, in lack of sufficient objective evidence to support this opinion. Symptoms in adults include nasal congestion/obstruction/impairment, nasal discharge (anterior/postnasal), facial pain/facial pressure, or hyposmia/anosmia [1]. In children, acute sinusitis is defined as an inflammation of the nose and paranasal sinuses with a sudden onset of symptoms that include at least 2 of the following: nasal congestion/blockage, impaired nasal breathing, mucopurulent nasal discharge, and coughing during the day or night [1]. Both adults and children need to be examined to rule out allergy (hay fever) and undergo the differential diagnosis between ARS and allergic rhinitis [1].

Refractory acute rhinosinusitis (RARS) refers to the presence of 4 episodes of acute rhinosinusitis per year, with distinct non-symptomatic intervals between episodes. Accordingly, each episode must meet the above-mentioned criteria for acute rhinosinusitis.

In terms of duration, EPOS2020 classifies ARS into acute viral aka common cold, acute post-viral, and acute bacterial rhinosinusitis (ABRS) [1].

A viral ARS is defined as an episode with the symptoms lasting less than 10 days. Acute post-viral rhinosinusitis refers to an increase in symptoms after day 5 or persistence of symptoms for more than 10 days and less than 4 weeks [1].

ABRS is characterized by the presence of at least 3 of the following symptoms and signs:

- Colored secretions
- Intense local pain
- Fever > 38 °C
- CRP/ESR increase
- Double sickening

It should be noted that in many cases ABRS is usually unilateral [1].

By definition, ARS lasts a few weeks at most and various classifications have been proposed in the literature. Previously, the term "subacute" was used to cover the period between acute (< 4 weeks) and chronic rhinosinusitis (> 12 weeks) [12]. The EPOS group, however, decided that a separate term to describe patients with prolonged discomfort is not necessary, since the number of these patients is limited and there are very little data to issue guidelines.

The above definitions derive from EPOS2020 and the unanimous opinion of the task force of the European Rhinologic Society [1].

The distinction between viral ARS and ABRS is mainly based on the pattern of illness and its duration, with viral ARS lasting less than 10 days [1]. The American Academy of Otolaryngology and Head and Neck Surgery defines ABRS as: a) symptoms/signs of ARS with no actual improvement for at least 10 days, or b) symptoms/signs of ARS that worsen within 10 days after an initial improvement (double sickening) [3]. The Canadian Academy guidelines define ABRS as symptoms persisting for 7 days and longer [13].

Acute exacerbation of chronic rhinosinusitis (AECRS—acute exacerbation of chronic rhinosinusitis) is also included in the context of acute infection and is described as an acute worsening of the pre-existing symptoms of CRS as the patient relapses into the initial status of disease automatically or after treatment [14, 15]. In the 2016 ICAR: RS (International Consensus Statement on Allergy and Rhinology: Rhinosinusitis), a definition of AECRS was proposed that included worsening nasal obstruction, congestion, nasal or postnasal discharge, facial pain, pressure or headache, alongside to a decrease in sense of smell [16]. This may be accompanied by endoscopic findings such as pus, crusting, edema, or polyps that establish the diagnosis of AECRS [16].

CRS (with or without nasal polyps) in adults includes the presence of two or more symptoms, one of which should be nasal blockage / obstruction / congestion or nasal discharge (anterior / posterior nasal drip), with/without facial pain/pressure; reduction or loss of smell; for 12 weeks or more [1].

## Diagnosis of ARS in Primary Care

ARS is characterized by an acute onset of a cluster of symptoms that include nasal obstruction, discharge, facial pain or pressure, and hyposmia or anosmia [1]. In primary care settings (and for epidemiologic studies), ARS is diagnosed based on symptoms, without the need for a detailed ENT examination or imaging and refers to the presence of accompanying symptoms for up to 12 weeks [4, 17, 18].

As previously mentioned, ARS is classified into 'acute viral rhinosinusitis', 'acute post-viral rhinosinusitis' and 'acute bacterial rhinosinusitis' [1]. In "acute viral rhinosinusitis", which is equivalent to common cold, the duration of symptoms is less than 10 days and it is usually a self-limiting condition that often will not lead the patient to the doctor [1]. "Acute post-viral rhinosinusitis" is characterized by exacerbation of symptoms after five days or their persistence beyond 10 days. In "acute bacterial rhinosinusitis" at least three of the following symptoms/signs are required: mucopurulent discharge, severe local pain, fever > 38 °C, elevated CRP (C – Reactive Protein) /ERS (Erythrocyte Sedimentation Rate) and "double sickening” [1].

ARS may be linked to other upper respiratory symptoms such as a sore throat, hoarseness of voice, and cough, as well as nonspecific systemic symptoms such as malaise, fatigue, and fever. Objective findings from nasal endoscopy, imaging, or puncture of the maxillary sinus are not required to diagnose uncomplicated cases. In adult patients with the initial suspicion of ARS based solely on symptoms, the prevalence of actual ARS through imaging, culture, aspiration or puncture confirmed ARS, is estimated to be 50% [16]. In such cases, anterior rhinoscopy is recommended to establish diagnosis, as it may reveal evidence of inflammation such as mucosal edema and secretions [1, 19]. Various diagnostic algorithms have been developed for ARS, but there are no prospective studies to support these data. ERS and CRP are inflammatory markers that are elevated in ARS, but are not routinely used in daily routine because of limited specificity.

ARS is on one hand underdiagnosed and on the other overtreated, thus missing out on opportunities to treat patients without the use of antibiotics [19–21]. Thus, the correct diagnosis of patients with ARS is vital for their proper treatment. In the majority of cases diagnosis is clinical, practically based on history and physical examination with a variety of associated symptoms and signs such as sneezing, malaise, fever, cough, nasal discharge, nasal obstruction, sore throat and headache [18, 19, 21]. However, many of them are non-specific and are also present in other infections or inflammations as well as in allergy exacerbations [22, 23].

The three main symptoms and signs that otolaryngology, rhinology and infection disease specialists agree are required to diagnose ARS include: 1) Up to 4 weeks of purulent nasal discharge, accompanied by 2) nasal obstruction, and facial pain/pressure/fullness, and 3) hyposmia / anosmia or both [24, 25]. Although there are no studies with a high level of documentation for these symptoms, they have nevertheless been proposed over many years by groups of experts. Therefore, eveh though nasal endoscopy is not necessary for the diagnosis, but anterior rhinoscopy is indicated to assess paranasal sinus drainage and other findings, such as mucosal inflammation and edema.

It is important to underline at this point that nasal obstruction alone without purulent nasal discharge is not sufficient enough for the diagnosis, whereas only facial pain or pressure without purulent nasal discharge does not establish a diagnosis of ARS [1]. The typical symptoms of allergic rhinitis, such as itching and watery eyes, should also be sought to differentiate ARS from an allergy or other headache disorders that may cause facial pressure and pain [22, 23].

## Diagnosis of ARS by the ENT Specialist

Uncomplicated cases of ARS are more likely to be seen in primary care settings as in GP offices. In some health systems however, as for instance in the Greek National Health System, with rather easy access to secondary and tertiary health structures and a largely developed private sector, patients with ARS can be examined by specialists. In general, the diagnosis can be made following the aforementioned criteria, but sometimes more detailed diagnostic tests can be applied, such as endoscopy and imaging, whereas immediate referral and/or hospitalization are mainly indicated for specific symptoms and deterioration of disease.

In research protocols, more data may be required for setting the diagnosis. Usually, a combination of symptoms is necessary, added to endoscopy, imaging, and culture from the middle meatus [25–27]. Ideally, the diagnostic criteria used throughout studies should be the same so that the results can be compared, but this is practically impossible.

In some settings such as ICUs, ABRS is common (risk factors include nasogastric tube, mechanical ventilation, supine position, and immunosuppression), and is often associated with -poor outcomes and in some cases, even sepsis [16, 24]. Consequently, in such cases, more aggressive diagnostic methods are required, since imaging can be useful for the diagnosis and sinus puncture by an experienced physician can provide important information regarding pathogens and options of treatment [28, 29].

Another special category of patients are immunocompromised patients since they are much more vulnerable to both ABRS and its complications and likewise, a more aggressive diagnostic approach is needed [30, 31]. Acute invasive fungal rhinosinusitis is a very serious disease with high morbidity and mortality requiring prompt diagnosis and treatment with open or endoscopic sinus surgery [32]. Diagnosis is usually histopathological, so early endoscopic evaluation is indicated followed by biopsy in cases where disease is suspected [32].

## Pathophysiology

ARS includes acute viral, post-viral and ABRS [1]. Besides the particular virus strain and its virulence, the severity and pathogenesis of ARS is highly dependent on the host and predisposing elements such as age, immunodeficiency, previous infection or immunization [16, 24, 33]. Other contributing factors include preexisting mucosal inflammation caused by exposure to allergens, pathogens or other environmental risk factors, as well as anatomical variations of the nose and paranasal sinuses [29, 33–35].

The pathogenesis and pathophysiology of ARS is not fully understood. This is mainly due to the lack of prospective clinical studies and basic research during the natural course of ARS, since in the literature, most of the studies cited are in vitro studies, using human tissue or cell lines from volunteers and experiments on animals and therefore the results have not yet been validated in cases with a naturally acquired viral infection and ARS [36–39]. The pathophysiology and inflammatory process of viral and postviral infection, as well as ABRS, may overlap significantly and the same applies to their clinical presentation.

Viral ARS (or common cold) is by definition an acute rhinitis caused by respiratory viruses such as rhinovirus (RV), respiratory syncytial virus (RSV), influenza virus (IFV), coronavirus (CorV), parainfluenza virus (PIV), adenovirus (AdeV) and enterovirus (EV) [1, 24]. The most common viruses isolated from adults with ARS accounting for approximately 50% of viral ARS are RV and CorV [40]. In children there is a broader phasma of viruses that are isolated, i.e. in addition to rhinoviruses and coronaviruses, RSV, PIV and AdeV are also often present [41, 42]. Depending on the geographic distribution, other viruses have also been isolated from patients with ARS, e.g. the human vocavirus [43]. With the advancement of medical technology and the development of highly sensitive methods for the detection of viruses, it is now possible to detect multiple viruses. To yet, it is rather difficult however to identify the exact viruses that cause or lead to an exacerbation of ARS in clinical practice.

The pathophysiology and the pathogenic mechanisms of postviral ARS have not been fully elucidated. A viral infection of the nose and paranasal sinuses causes many changes, such as mucosal infiltration by various inflammatory cells or activation of others, as well as impairment in innate and humoral immunity, which increase the risk of bacterial infection [37–39, 44]. Only a small percentage of patients with ARS will eventually develop ABRS suggesting that postviral ARS is not an indication of the development of a bacterial infection [1].

According to EPOS2020, ABRS is an uncommon complication of viral upper respiratory tract infections causing mucosal damage and bacterial 'super-infection' [1]. Disruption of mucosal function due to viral infection is probably a major cause of super- or secondary bacterial infection [44]. Bacterial and fungal infections are usually accompanied by viral infections, such as in the common cold (RV-infection) and CRS [1]. Streptococcus pneumoniae, Hemophilus influenzae and Moraxella catarrhalis are the most common pathogens in rhinosinusitis [45]. In another study, in cell cultures with RV-1b infection, a mechanism involving virus-induced release of IL-6 and IL-8, and overexpression of ICAM-1, can promote Staphylococcus aureus penetration [46]. Research in primary human respiratory epithelial cells has found that RV infection can promote the expression of cell adhesion molecules and bacterial adhesion itself [47]. Furthermore, in nasal epithelial cells (NECs) from the nasopharynx of patients with RV, TNF-α (tumor necrosis factor α) expression was increased after Aspergillus infection [48].

Viral infection of the nasal mucosa can initiate a cascade of inflammation, which at the same time is involved with symptoms of common cold, forming the basis of the immune response [49, 50]. "Neutralization" of the virus leaves dead epithelial and inflammatory cells, which add to the pathology of ARS [51, 52]. Such transcriptional alterations in epithelial cells can significantly affect the immune response and homeostasis, increasing the pathology and complications of respiratory infections [53, 54] creating suitable conditions for the development of secondary bacterial infections (Staphylococcus aureus and Streptococcus pneumoniae), that represent another aggravating factor [55]. Nonetheless, respiratory viruses induce the production of type I interferons (IFN), inhibiting the recruitment of circulating neutrophils and macrophages to the lung after bacterial challenge 49,55,56). They also promote the differentiation of primitive T cells into T17 helper cells (TH17) or other TH types (such as TH1 and TH2 cells), thus enhancing the susceptibility of the host to secondary bacterial infections [49, 56]. The ability of immune cells, such as macrophages, to kill bacteria can be inhibited by the production of interleukin-10 (IL-10) from influenza virus-specific T-cells [49, 56]. Finally, the interaction of immune cells — such as macrophages, neutrophils and natural killer cells (NK) — with the influenza virus decreases the ability of these cells to neutralize bacteria [49, 56].

S. pneumoniae infection is usually associated with exacerbation of viral infections and research has shown that the influenza virus has the ability to alter the gene expression of S. pneumoniae by augmenting the dispersal of biofilms throughout the nasal mucosa [57, 58]. As other respiratory viruses induce similar antiviral activity in the nasal epithelium, these viruses can disperse S. Pneumoniae in the airway mucosa as well [57, 58].

The nasal mucosa consists the main point of entry to the human body for irritants, allergens and common pathogens, as well as respiratory viruses. At the same time, it represents a target organ for the reproduction of viruses of the respiratory system and furthermore for the activation of cellular and humoral immunity mechanisms. Epithelial cells of the nasal mucosa express several receptors that recognize specific viruses, such as intercellular adhesion molecule-1 (ICAM-1), toll-like receptor 3 (TLR3), α−2,3-linked sialic acid-containing receptor (α−2,3-SA)/α−2,6-SA), retinoic acid inducible gene 1 (RIG-1, also known as DDX58), as well as MDA4—Melanoma Differentiation-Associated Protein (also known as IFIHI—Interferon Induced with Helicase C Domain 1) [59].

At the early stages of disease, the virus enters the cells of the nasal mucosa with the help of special receptors leading to expression and replication of viral strains that is achieved within hours of infection [60]. One of the most important defense mechanisms of the nasal muco-ciliated epithelium is the muco-ciliated clearance and the function of the cilia [38, 44, 61]. There are data supporting the significant and longstanding deregulation of the clearance capability of the nasal mucosa that can last up to 32 days in patients suffering from common cold [38, 61, 62]. This damage includes both a reduction in the population of of ciliated cells and a change in the frequency of cilia movement [63, 64]. Other studies have further proven that the ability of epithelial cells to produce cilia is significantly reduced after persistent viral infections leading to a continuous loss of fimbriae and to their structural derangement (e.g. in the cytoplasm and mitochondria) [38, 44, 61, 62]. RSV has been preferably linked to infecting ciliated cells in human primary nasal epithelial cells since portions of RSV proteins (F and G) were transported from the cilia 24 to 48 h after infection, leading to extensive loss of cilia after five days [61, 62]. Finally, infections involving the influenza virus infection, lead to apoptosis, i.e. cell death of epithelial cells and ciliated cells, thus affecting the function of the cilia [61, 63].

## Treatment

Currently, multiple systematic reviews and meta-analyses have addressed important parts of the management of acute viral ARS. For postviral rhinosinusitis and ABRS, new systematic reviews and meta-analyses have also recently been performed and we discuss some of the most interesting aspects.

## Traditional Medicines

### Antibiotics

Eleven randomized controlled trials conducted after EPOS2012 compared antibiotic treatment versus placebo in patients with symptoms of common cold. Indicatively, a total of 1047 participants who received antibiotics for treating common cold had no better results to their outcome or persistence of complaints compared to those who received placebo (RR response rate 0.95, 95% CI confidence interval 0, 59 to 1.51) [1]. Adult participants who received antibiotics had a significantly higher risk of adverse events than those who received placebo (RR 2.62, 95% CI 1.32 to 5.18) (random effects), whereas the same was not found in the pediatric population (RR 0.91, 95% CI 0.51 to 1.63) [1, 65]. The conclusion was that there is no evidence to support the use of antibiotics for common cold or for acute purulent rhinitis in pediatric or adult population [1, 63, 64]. In contrast, it is well known that antibiotics can have a large amount of side effects when prescribed in adults for common cold and regardless of age when prescribed for purulent rhinitis, therefore antibiotic prescription for these pathologies is not recommended [1, 63, 64].

### Nasal Corticosteroids

The anti-inflammatory effect of nasal corticosteroids is considered to have a beneficial effect on common cold symptoms [1]. In 2013, a Cochrane review compared nasal corticosteroids with placebo and standard treatment measures for the common cold in children and adults [65]. A total of 353 patients were included of which, two trials compared nasal corticosteroids with placebo, while one trial compared nasal corticosteroids with usual symptomatic treatment [1, 65]. In the two placebo-controlled studies, no benefit from the use of nasal corticosteroids was depicted in terms of length or intensity of complaints [1, 65]. The conclusion being that current evidence does not justify the use of nasal corticosteroids for symptomatic relief of common cold.

### Oral Corticosteroids

There are a few studies have been published investigating the use of oral corticosteroids as adjunctive therapy for the treatment of ARS. Some of them, such as Gehanno, Ratau et al., support the use of systemic steroids and especially methylprednisolone and betamethasone to treat ARS-related symptoms, especially facial pain. However, later studies with more rigorous methodology and without the use of antibiotics as adjunctive therapy failed to document an important relief for patients diagnosed with ARS who received prednisone [66].Additionally, a Cochrane meta-analysis failed to demonstrate a significant benefit from the use of systemic corticosteroids in ARS, despite the fact that over a thousand patients were included in these studies. However, criticism exists regarding the diagnostic criteria for ARS followed in all these studies.

### Paracetamol

A Cochrane literature review found that paracetamol can improve nasal obstruction and nasal secretions; however it does not appear to have an effect on other symptoms of the common cold, including pharyngalgia, malaise, wheezing and coughing [67].

### Decongestants

In 2016 a Cochrane review was conducted in terms of efficacy, short or long-term safety of topical and/or oral decongestants, used to relieve symptoms of the common cold in adults and children [68]. A total of 15 trials and 1838 patients were included with the authors concluding that, with the current data, courses of treatment with decongestants may have little positive effect on the feeling of nasal obstruction in adults suffering from common cold [68]. Due to the limited number of studies including a newer nasal decongestant, no conclusions were drawn regarding the efficacy of oral versus topical decongestants without raising the risk of side effects in adults on the short term.

### Antihistamines

Antihistamines are used in ARS to reduce nasal secretions. However, it has been reported that the increased viscosity of secretions from the use of antihistamines could cause a decrease in mucociliary clearance and predispose to ABRS. Nevertheless, systematic studies have failed to produce a benefit from the use of antihistamines in adults for the treatment of ARS [1, 69, 70]. However, Braun and colleagues, in a randomized controlled trial in patients with ARS and confirmed allergic rhinitis showed an improvement in patients' symptoms when loratadine was added to antibiotics for the treatment of ARS [69].

### Nasal Washes

Saline nasal irrigations are often used as an adjunctive treatment for URTI symptoms. In 2015, a review of five RCTs (randomized control trials) was published, where nasal douching was evaluated against nasal sprays but not placebo. The primary objectives of the studies differed considerably and it was not possible to reach firm conclusions, however, the therapeutic strategies under evaluation did not differ drastically in controlling nasal symptoms. A study that followed, including a larger number of children, depicted a significant improvement of nasal congestion and nasal discharge, as well as a significant reduction in the use of decongestants in the nasal rinse group [1]. Mild nasal discomfort and/or irritation were the only adverse effects reported by a minority of patients and the authors concluded that saline nasal washes have a positive effect on common cold symptoms [1, 71].

### Combinations of Antihistamine—Decongestant – Analgesic

Antihistamine-decongestant-analgesic combinations are a common treatment in URTI. The evaluation of the effectiveness of these combinations in terms of shortening duration and relieving symptoms was carried out in a review of 27 studies including adults and children. Fourteen trials were done with an antihistamine-decongestant combination, two with antihistamine-analgesic, six with analgesic-decongestant and five with antihistamine-analgesic decongestant trials [72]. In 21 trials the control intervention was placebo and in six trials an active substance. Evidence from the systematic review proposed that antihistamine-analgesic-decongestant combinations have a positive effect in adults and older children but there was no evidence of effectiveness in young children and nevertheless these benefits need to be weighed against the risk of adverse effects [1, 72].

## Μedicines of Plant Origin

The effectiveness of herbal therapy for the treatment of common cold has been investigated through studies including a sufficient sample of patients [1]. Up till now however, no systematic review of the literature of the use of herbal medicine for common cold has been conducted. A recent review by Koch and colleagues included patients with symptoms and signs suggestive of both viral ARS and postviral ARS, as well as a few patients with ABRS therefore leading to none conclusive results [8].

Existing literature reports 2 studies with a total of 302 patients using cineol, an extract of eucalyptus oil with anti-inflammatory properties [10, 73]. In the first study, patients received cineole or placebo, and in the other, cineole or a different herbal preparation to treat viral ARS [10]. Both studies, after 7 days of treatment, showed a larger decline in total symptom scores, individual symptom scores and improvement in rhinoscopy in the cineole group compared to the control group [10, 73]. Two more studies were conducted evaluating the efficacy of andrographis paniculata SHA-10 extract for five days in the first, and Kan Jang (standardized andrographis paniculata SHA-10 extract) and eleutherococcus senticosus extract in the later, again for the same amount of time [9, 74]. The objective was to study the prevalence and severity of signs and symptoms in 158 and 200 patients respectively with common cold compared to placebo. The results revealed significant improvement in symptoms for the andrographis paniculate group in both studies, where no side effects were reported [9, 74]. In addition, Hawkins and colleagues published a systematic review on the potential use of black elderberry (sambucus nigra) in common cold with however heterogeneous studies of patients with both influenza and common cold included in this review [75]. The part of the study that evaluated the patients with common cold showed no statistically significant improvement over placebo [75].

BNO1016 is an extract of five herbal medicines (gentian root, primrose flower, sorrel herb, elder flower, verbena) presenting antimicrobial and antiviral activity [76, 77]. Four studies have described the effectiveness of BNO1016 in patients with symptoms of common cold. Jund et al. randomized a total of 600 patients with viral ARS into 2 double blinded placebo-controlled trials of 2 groups, thus BNO1016 extract and placebo. Patients receiving BNO1016 extract had a statistically better response to treatment, a significant improvement in their SNOT-20 questionnaire, and similarly in scores regarding cardinal symptoms, by the 14th day of treatment, without serious adverse events being noticed in either study [77]. BNO1016 action versus an antibiotic was evaluated in 64 patients with common cold with the BNO1016 group reporting faster relief of headache, reduction of nasal blockage, secretions and hyposmia and a similar improvement in rhinoscopy findings [77]. Finally, in another study, BNO1016 syrup when administered to a group of 184 children, aged 6 to 11 years, was found to be superior to saline washes and symptomatic medication, on days 5 to 8, when symptom assessment was addressed [78].

Neubauer and colleagues studied the action of BNO1016 in post-viral ARS, in 160 patients with symptoms and signs of postviral rhinosinusitis that were randomized into 2 treatment groups [79]. In particular, one group received BNO1016 as adjunctive therapy along with doxycycline and oxymetazoline, and the control group received placebo in combination with the antibiotic and a decongestant. A greater proportion of patients in the BNO1016 group presented with a complete resolution of symptoms (60.3% vs. 25.0%· p = 0.0002) and improvement in radiographic findings after 14 days of treatment (84.0% vs. 68.4% p = 0.02) in comparison to the placebo group. BNO1016 showed a small improvement in nasal obstruction and mucosal edema, however there was no difference in complaints of nasal patency, nasal discharge and headache [79]. In another study, BNO1016 was compared to treatment with fluticasone furoate, in a group of 60 patients with ARS that were randomized to BNO1016 or intranasal fluticasone furoate spray for 14 days [80]. In this study, no statistically significant differences were found between the 2 groups, probably due to a type II statistical error [81].

On the contrary, data regarding CRS and herbal medicines are limited. In a randomized placebo-controlled study specific herbal extract showed superiority over placebo in 929 patients suffering from CRS [76]. A post-hoc subgroup analysis in patients whose disease was diagnosed by a specialist revealed a definite treatment effect in means of duration and intensity leading the authors to conclude that this extract can safely be administered in patients with CRS [76]. In another study, 40 patients with CRSsNP were randomized in 2 therapeutic arm groups; herbal extract orally, and nasal corticosteroid spray for one month respectively. Patients who received herbal extract had lower TNSS (Total Nasal Symptom Score), and TES (Total Endoscopic Score) than their counterparts. Moreover, no adverse events were noted [81].

## What are Medicinal Plant Treatments?

Every medicine that exclusively contains as its active ingredient one or more plant substances, or one or more herbal preparations, or a combination of one or more plant substances with one or more herbal preparations, is defined as a medicine of plant origin [82]. Plant substances refers to the whole part of the plant, shredded or cut plants, parts of plans, algae, fungi and lichens, which are unprocessed, usually dried or sometimes fresh [82]. Exudates, which have not undergone processing, are also considered medicinal treatments of plant origin [82]. Plant substances are precisely identified by the part of the plant used and the botanical name according to the binomial system. Herbal preparations are obtained by subjecting plant substances to processing such as extraction, distillation, compression, fractionation, purification, concentration or fermentation. Herbal preparations include shredded or powdered plant substances, tinctures, extracts, essential oils, juices and processed exudates [82].

## What is Phytoneering?

The use of medicinal plants as a practice to treat a wide range of symptoms has been established over the centuries in several paradigms. The treatment of diseases with the use of medicinal plant treatments (phytotherapeutics) is gaining more and more followers in recent years. An increasing number of people nowadays prefer using effective substances with limited side effects, which have been derived from nature, as an alternative to synthetic substances for the treatment of various conditions. Knowledge of traditional medicine and modern practices of processing botanical agents, form the basis of modern phytotherapy.

The use of innovative pharmaceutical techniques and processes plays a vital role in the research and production of herbal medicines. This production process is named phytoneering and it refers to a special link between nature and plants (phytos) on one hand, and science and technology on the other (mechanics) [80]. At the same time, it represents an international orientation of turning the highest quality of standards for herbal medicines into a reality. A key element of this concept is the continuous optimization and certification of all production processes which starts with the selection of the seeds, continues with the cultivation of the plants, the isolation of the active substances from the parts of the plant and the final production of the medicinal preparation [80, 83].

In phytoneering, every phase of production is based on scientific efficiency and cutting-edge technical methods [80, 83]. The production as a whole is adapted to fulfilling the special requirements necessary in the processing of herbal substances. Continuous quality control measures ensure the manufacturing process, from the cultivation of the plants to the release of the medicinal product and delivery to pharmacies [80, 83]. The high concentration of active chemical compounds in the final product is the result of this optimized high-tech production process, which is the foundation basis of phytoneering [80].

Phytoneering also ensures that the company's herbal medicines are tested for efficacy and safety [80, 83]. Herbal medicines are tested in preclinical and clinical studies that meet the same strict criteria for synthetically produced prescription substances. Thus, specific pharmaceutical companies carry out randomized studies according to GCP (good clinical practice) and in this way excellent efficacy documentation is obtained. However, the optimization of the production process also requires up to date knowledge of the underlying mechanisms [80, 83]. This is a basic axis which is supported by the conduction of studies in institutes and universities [80, 83].

Therefore, due to the systematic application of the phytoneering concept, herbal preparations differ in quality and active substances, with these modern herbal medicines being at least on par with synthetically produced medicines, in terms of quality, safety and efficacy, but having significantly less unwanted side effects [80, 83]. In conclusion, phytoneering is the link between traditional botanology and modern medicine [80, 83].

The composition of the natural dry extract BNO 1016, in terms of herbs, is identical to that of BNO 1011, however, the dry extract BNO 1016 contains comparatively higher concentrations of the active ingredients, since it was prepared with a 59% ethanol extract (V/V) (with a drug: extract ratio of 4.2:1) [84–86]. Both formulations were manufactured using a validated GMP manufacturing process, applying comprehensive specifications and standardized production processes, to guarantee high product quality from batch to batch [84–86]. The quality of herbal medicines is determined according to the relevant EMA (European Medicines Agency) guidelines for herbal medicinal products.

## Conclusion

Phytoneering is an innovative pharmaceutical technique in the field of research and production of herbal medicines [84, 85]. Herbal extracts produced with phytoneering differ in quality and active substances but in terms of quality, safety and efficacy, these modern herbal medicines can be comparable to synthetically produced medicines, having much less unwanted side effects [80]. Specific herbal medicines such as BNO1016 extract, cineole and andrographis paniculata SHA-10 extract, have a significant effect on common cold symptoms without significant side effects. In post viral sinusitis, and CRS, BNO1016, may be a good alternative. Little by little herbal extracts gain their place among traditional medicines in Western world. Phytoneering seems to be Cinderella’s glass slipper!

## Data Availability

No datasets were generated or analysed during the current study.
